# Rheological and technological characterization of red rice modified starch and jaboticaba peel powder mixtures

**DOI:** 10.1038/s41598-021-88627-4

**Published:** 2021-04-29

**Authors:** Raphael Lucas Jacinto Almeida, Tamires dos Santos Pereira, Renata Duarte Almeida, Ângela Maria Santiago, Wanda Izabel Monteiro de Lima Marsiglia, Elizabeth Harumi Nabeshima, Líbia de Sousa Conrado, Rennan Pereira de Gusmão

**Affiliations:** 1 Department of Chemical Engineering, Federal University of Campina Grande, Campina Grande, Brazil; 2 Department of Process Engineering, Federal University of Campina Grande, Campina Grande, Brazil; 3 Department of Food Engineering, Federal University of Campina Grande, Campina Grande, Brazil; 4 Department of Chemistry, State University of Paraíba, Campina Grande, Brazil; 5 Institute of Food Technology, Cereals and Chocolate Technology Center, Campina Grande, Brazil

**Keywords:** Chemical engineering, Biotechnology

## Abstract

Properties of modified starch and its interaction with functional raw materials are of great interest to the food industry. Thus, this study aimed to evaluate the rheological and technological characterization of starches modified by the action of the enzymes α-amylase and amyloglucosidase and their mixtures with jaboticaba peel powder. The parameters of firmness, gumminess, and final viscosity of starches paste increased, and the tendency to setback was reduced with the addition of jaboticaba peel powder. Starches and mixtures presented shear-thinning behavior. The addition of jaboticaba peel powder to starches increased water, oil, and milk absorption capacity, while syneresis remained stable over the storage period. The addition of jaboticaba peel powder had a positive effect on native and modified starches' rheological and technological properties, qualifying it as an alternative for developing new functional food products.

## Introduction

A wide range of native starches with profoundly different functionalities is required to meet market needs^1,2^. However, native starches have technological limitations^3^. Thus, there is substantial interest in modifying their structures to improve properties and increase starch application^4^. Enzymatic hydrolysis is considered the most promising approach offering more environmentally friendly processing since no solvents or chemical reagents are used^5^. Partial enzyme treatment modulates the granule starch's external macromolecular structure, thereby producing a slow-digesting starch^6^. Additionally, alterations of the starch molecular structure must obtain the desired viscoelastic properties and develop a more palatable food and increase shelf-life stability^7^.

Besides, new starch sources specifically targeted to have a specific structure and functionality are required to meet market needs. This challenge may require new ingredients to help meet those market needs^8^. Rice (*Oryza sativa* L.) is part of the essential human diet and is consumed worldwide. Rice is composed mainly of carbohydrates as starch, comprising 90% of its total dry weight^9,10^. According to the study by^4^, native red rice starch comprises 18% amylose and 64% amylopectin. However, starches from different sources exhibit specific physicochemical, textural, rheological, and digestive properties of each particular plant^11,12^. Starch processing into food products is often performed at relatively high temperatures. These thermal treatments alter the starch rheological properties that influence several textural properties, nutritional quality, and ultimately, starch products' acceptability^13,14^.

Starch is the main ingredient in many foods and significantly contributes to the textural properties of the food. Moreover, starch can be used as a thickener, stabilizer, gelling agent, and water-retaining agent for many industrial applications^15^. However, the low shear and thermal resistance while cooking and the syneresis of native starch preclude its use in specific food industry applications^16,17^. The peel of the jaboticaba (*Myrciaria* *cauliflora*) fruit is a food byproduct with nutritional and properties that can be explored. It has recently attracted researchers' attention who found high soluble and insoluble fibers, mineral salts, and phenolic compounds. Among the phenolic compounds, it is highlighted the high contents of anthocyanins, flavonoids, ellagitannins, and condensed tannins. These bioactive molecules have a powerful antioxidant effect that plays an essential role in the body's defense system inhibiting free radical scavenging^18,19^.

Dietetic fibers, either soluble or insoluble, are edible polysaccharides that cannot be hydrolyzed by the enzymes present in the human gastrointestinal (GI) tract^20^. The ingestion of insoluble fibers can cause a satiety sensation since it absorbs water and thus increases its size during the GI tract, which can cause pronounced effects on the intestinal transit and feces volume^21^. Some products, available in the Brazilian market, use fruit byproducts claiming health benefits to human health because of their high fiber content^20^. Dietetic fibers present in jabuticaba peel can increase the nutritional value of different foods, including breakfast cereals, bakery products, dairy, pet food, and can be used to formulate farmaceutical products using slow delivery of antioxidants^18^.

Therefore, the inclusion of such fruit byproducts can have a positive and synergic effect on starchy foods since they can increase the dietary and bioactive contents^22,23^. Besides the nutritional improvement, the inclusion of fruit byproducts can improve the starch's technological properties. The present work will provide helpful information regarding the rheological and technological characterization of a mixture between a red rice starch modified by hydrolysis with two enzymes (α-amylase and amyloglucosidase) and powdered jaboticaba peels at 10% wt.

## Materials and methods

### Extraction of native starch from red rice

The red rice grains (*Oryza Sativa*) were purchased in the local market. Starch was extracted by the method previously described elsewhere with adaptations^24^. Briefly, the red rice was firstly immersed in a sodium metabisulfite solution (0.2%) (Sigma-Aldrich, São Paulo, Brazil) in a ratio of 1:2 (w/v) for 48 h and then rinsed in running water. The treated red rice was then homogenized along with distilled water at a ratio of 1:2 (w/v) using an industrial blender (Manufacturer Kohlbach, model KM42A) for 5 min and then filtered to obtain the starch suspension. The filtration residue was again homogenized to increase the process yield. The starch suspension was decanted for 24 h in a refrigerated environment at 7 °C to avoid enzymatic or fermentative action during the settling process. The decanted starch was submitted to convective drying at 50 °C until constant weight. Finally, the native starch was vacuum packed and stored at room temperature (25 ± 3 °C) and kept away from light exposure to avoid bioactive compounds' losses.

### Enzymatic modification of starch

The commercial enzymes α-amylase and amyloglucosidase were kindly supplied by (CBB–Biomassa e Bioprocessos, LTDA, São Paulo, Brazil). Both products had their enzymatic activity determined. First, the native starch (SN) was hydrolyzed with the α-amylase enzyme producing the formulation (MS1). Another hydrolysis was performed with the amyloglucosidase enzyme, thus acting on the α-amylase modified starch mass in the first hydrolysis and producing the formulation (MS2). For the hydrolysis of native starch by α-amylase, the process conditions were: enzymatic charge (3UE g^−1^), mass: volume ratio (2 RZ), pH 8, time 18 h, starch mass 4 g, 60 °C, and stirring at 150 rpm.

For the starch hydrolysis by the amyloglucosidase, the process conditions were: enzymatic load (3UE g^−1^), mass: volume ratio (2 RZ), pH 6, time 3 h, starch mass 4 g, temperature 60 °C, and stirring at 150 rpm. Both processes were performed following the optimal hydrolysis conditions for this material, according to the study by^4^. The percentage of hydrolysis characterized the hydrolysis process. The absorbance measurements were taken at the wavelength of 540 nm^25^.

### Production of Jaboticaba peel powder

The jaboticaba fruits were purchased in the local market and selected, washed, and sanitized with sodium hypochlorite in a solution (200 mg L^−1^) of free chlorine for 15 min and then rinsed with running water. The fractions were separated (pulp, peel, and seed) by manual pulping and submitted to the convective drying process, carried out in an oven with air circulation (Marconi—MA048) temperature 50 °C and an airspeed of 1.0 m s^−1^ for 24 h. The jaboticaba peels were crushed in an industrial blender (Kohlbach—KM42A) within 3 min. The sample was packaged in a polyethylene package with a zip lock under the light shelter.

#### Characterization of jaboticaba peel powder

The methodologies used were: moisture (method 934.06), ash (method 940.26), lipid (method 948.22), protein (method 920.152)^26^, crude fiber (FB), in neutral and acid detergent^27^, total carbohydrate content was calculated by difference. Water activity (a_w_) was determined using the Decagon Aqualab CX − 2 T device at 25 °C. Anthocyanins and flavonoids^28^, total carotenoids (lycopene)^29^, total tannins^30^ methodology with adaptations, total phenolic compounds^31^. The antioxidant activity was determined by the ABTS^•+^ method using the method proposed by^32^, with modifications made by^33^. The antioxidant activity by DPPH^•^ was performed using the methodology described by^34^, with adaptations. For both analyzes, distilled water was used as the extractive solvent.

### Mix production

The powdered jabuticaba peel (JPP) was added to all starch samples (SN, MS1, and MS2) at the same concentration of 10%. Therefore three new formulations were composed of JPP at 10% and 90% (w/w) of one of the starch samples (SN + JPP, MS1 + JPP e MS2 + JPP). The batch mixing process used the diffusion mechanism, using a non-segregating Y-mixer (Model, Manufacturer, Location) with a stainless steel chamber and a residence time of 10 min. The mixture was considered complete when both materials were randomly mixed against each other.

### Pasting and rheological properties

#### Pasting properties analysis

Pasting properties were determined with a Rapid Visco Analyzer (RVA-4500 model, Perten Instruments, Warriewood, NSW, Australia)^35^. Standard profile STD1 supplied with the instrument was used with 3.0 g of one of the mixtures (corrected to 14% moisture content) with 25 mL of deionized water. The total cycle time was 13 min. Peak viscosity (PV), viscosity at trough (also known as minimum viscosity, MV), and final viscosity (FV) were recorded. Breakdown (BD, which is PV minus MV) and setback (SB, which is FV minus MV) were calculated using the software Thermocline for Windows, version 3 provided with the instrument.

#### Rheology measurements and modeling

The six starch: water suspensions (1:10 w/v) were kept at 80 °C for 30 min for complete gelatinization and then cooled to temperatures of 70 °C and then 25 °C. The temperature was maintained using a thermostatically controlled water bath connected to a jacketed vessel. Viscosity and torque measurements were performed using a Brookfield viscometer (DV-II + PRO model, Brookfield Engineering, Laboratories Inc., MA, USA) with spindles RV06 and RV07 at nine spindle speeds (50, 60, 70, 80, 90, 100, 120, 135, 140, 150, 160, 180 and 200 rpm). Measurements were performed at 70 ± 2 °C and then at room temperature (25 ± 1 °C). Shear stress and shear rates were calculated by the method of^36^. The experiments were performed in triplicate, and the results were analyzed with the aid of Origin software version 8.0^37^. Results were modeled using the Ostwald-de-Waele (Power Law), Eq. (1).

1 $$\tau =K{(\gamma )}^{n}$$

where τ is the shear stress (Pa), K the consistency index (Pa.s^n^), γ is the shear rate (s^−1^), and n the flow behavior index^38^.

### Technological characterization

#### Texture analysis

Six suspensions were produced using a ratio of powder to the water of (1:10 w/v). After complete mixing, each suspension was heated at 80 ± 1 °C for 30 min. The obtained gels were poured into a cylindrical container (diameter 50 mm, height 40 mm) and stored at 6 °C. After 24 h, the samples were taken from refrigeration and placed at room temperature.

The texture profile was determined using the TA-XT PLUS texture analyzer (Scarsdale, NY; Stable Micro Systems, UK). Instrument settings were compression mode, trigger type, auto 5 g; pretest speed, 2.0 mm s^−1^; post-test speed, 1.0 mm s^−1^; test speed, 1.0 mm s^−1^; compression height, 40 mm; strain, 40%; interval between two compressions, 5 s; compression times, 2 s. Each sample was measured twice at room temperature (25 ± 1 °C), and the final result was the average of both tests. The firmness, cohesiveness, gumminess, and adhesiveness values were calculated from the force–time plots obtained^39^.

#### Water and oil retention

One gram of each of the six starch + JPP samples was combined with 10 mL of water or sunflower oil in centrifuge tubes. Slurries were kept at 24 °C and occasionally stirred with a glass rod over a 30 min period and then centrifuged at 15000 g for 15 min. The volume of decanted supernatant fluid was measured, and the amount of water or oil retained per gram of sample was calculated according to Eq. (2). ^40^.

2 $$ AC({g}{100^{ - 1}}) = 1 - \frac{{{M_{dry}}}}{{{M_{wet}}}}{{ \times 100}}$$

where: AC is the absorption capacity expressed in (g 100 g^−1^); M_Wet_ is the mass of the sample moist after centrifugation (g); M_dry_ is the mass of dry starch (g).

#### Milk absorption index

The milk absorption index was measured using a technique developed for cereals^41,42^. Briefly, 2.5 g of each of the six powder samples was suspended in 30 mL of milk at room temperature for 30 min and softly stirred. After this time, the mixture was centrifuged at 3000 g for 15 min. The supernatant was decanted into an evaporation dish of known weight. The milk absorption index is the gel's weight obtained after removing the supernatant per unit weight of original dry solids. The final value was determined by Eq. (3).

3 $$ MAI({g}100{g^{ - 1}}) = \frac{{{M_{CR}}}}{{{M_S} - ({M_{ER}}{{ \times 3)}}}}{{ \times 100}}$$

where, M_ER_ = weight of sample evaporation residue (g); M_S_ = sample weight (g), dry basis; M_CR_ = weight of centrifugation residue (g).

#### Syneresis measurement

Syneresis index capacity was determined according to the methodology proposed by^43^, with modifications performed for all gel formulations (SN, MS1, MS2, SN + JPP, MS1 + JPP, and MS2 + JPP) and followed for 28 days at 7-day intervals for each reading, as follows: (D0, D7, D14, D21, and D28).

Three days after the gel preparation and storage at 6 °C, the samples (15 g) were centrifuged at 3000 *g* for 10 min under refrigeration at 6 °C, in duplicate. The water mass separated from the gel network during centrifugation, divided by the initial gel weight and multiplied by 100, according to Eq. (4), calculated the percentage of syneresis.

4 $$Syneresis({g}100{g^{ - 1}}) = \frac{{M_w}}{{M_i}}{{ \times 100}}$$

M_w_ is the water's weight separated from the gel after centrifugation (g), and M_i_ is the initial gel weight (g).

### Statistical treatment

All experiments were executed in triplicate, and the results were submitted to analysis of variance (ANOVA) at 5% of probability. The significant qualitative responses were submitted to the Tukey test adopting the same level of 5% of significance. All statistical analyses were performed using Statistica software version 7.0^44^.

## Results and discussion

### Enzymatic hydrolysis

The result of enzymatic hydrolysis with α-amylase (MS1) expressed in the percentage of hydrolysis was 5.64%. The value obtained for reducing sugar at the second hydrolysis with amyloglucosidase (MS2), after the initial hydrolysis with α-amylase, corresponding to a percentage of hydrolysis 8.63%, totaling 14.27% of hydrolysis with two enzymes. According to Almeida et al., 2019^4^, the ash content ranged from 0.45 to 0.60 g 100 g^−1^, the lipids content ranged from 0.06 to 0.09 g 100 g^−1^ proteín content ranged from 0.60 to 0.74 g 100 g^−1^ and did not significantly change after the enzymatic treatment.

Partial or total hydrolysis of starch in its subunits (glucose) is a standard process for preparing starch products and controlling the starch content in different plant matrices. This hydrolytic process generally involves enzymes capable of disrupting the polysaccharide's glycosidic bonds under mild conditions. This process is based on two enzymes' complementary action: alpha-amylase followed by amyloglucosidase, responsible for the cleavage of glycosidic bonds (α-1,4 for alpha-amylases and α-1,4/α-1,6 for amyloglucosidase), respectively, which generates more fragments and, consequently, more non-reducing ends^45–47^.

In the present work, the red rice starch did not undergo a heat pre-treatment, gelatinization, and paste formation before or during the enzymatic process; the substrate was raw starch in the powder form. Therefore, only partial hydrolysis was obtained, which can be confirmed with the data presented in the study carried out by^4^, since the purpose of the present work is the rheological and technological analysis of the modified starch produced previously.

### Characterization of jaboticaba peel powder

Table 1 presents the data from the centesimal analysis, physicochemical and bioactive compounds for jaboticaba peel powder (JPP).

**Table 1:** Results of the centesimal, physicochemical and phytochemical characterization of the jaboticaba peel powder.

Parameters	Jaboticaba peel powder (JPP)
Moisture (g 100 g^−1^)^a^	9.46 ± 0.61
Ashes (g 100 g^−1^)^a^	3.06 ± 0.09
Lipids (g 100 g^−1^)^a^	1.22 ± 0.12
Protein (g 100 g^−1^)^a^	6.13 ± 0.62
Crude Fiber (FB) (g 100 g^−1^)^a^	4.36 ± 0.55
Neutral detergent fiber (NDF) (g 100 g^−1^)^a^	12.11 ± 0.41
Fiber Acid Detergent (FDA) (g 100 g^−1^)^a^	12.32 ± 0.37
Carbohydrate* (g 100 g^−1^)^a^	51.77 ± 0.48
Water Activity (a_w_)	0.344 ± 0.21
Anthocyanins (mg 100 g^−1^)	83 ± 1.95
Flavonoids (mg 100 g^−1^)	108 ± 5.51
Total Carotenoids (μg g^−1^ lycopene)	18 ± 1.12
Total Tannins (% eq tannic mL^−1^)	264 ± 6.51
Total Phenolic Compounds (Aqueous Extract) (mgGAE 100 g^−1^)	2549 ± 355.12
Antioxidant activity (ABTS^•+^) (µmol Trolox g^−1^)	403 ± 112.48
Antioxidant activity EC50 (g g^−1^ DPPH^•^)	180 ± 20.34

^**a**^Dry basis.*Calculated by the difference.

The moisture value found for jaboticaba peel powder was 9.46 g 100 g^−1^, which is in accordance with the Brazilian Legislation requirement for the dust/flour identity and quality standard, which recommends values in the range of 5 to 15 g 100 g^−1^^48^. In^49^ while characterizing jaboticaba flour found a moisture content of 14.45 g 100 g^−1^. In^50^ found a value of 15.33 g 100 g^−1^ for the jaboticaba peel flour.

Ash content was 3.06 g 100 g^−1^, consistent with those determined elsewhere^51^: found higher values, 4.23 g 100 g^−1^ for forced-air-dried jaboticaba peel, and 3.52 g 100 g^−1^ for freeze-dried peel, respectively. In^52^ found ash content values of 3.89 g 100 g^−1^ for JPP obtained after convection drying at a temperature of 60 °C.

The value found in the present work for the lipid content of jaboticaba peel powder (JPP) was 1.22 g 100 g^−1^, lower than the value found by^51^ of 1.72 g 100 g^−1^ for the lyophilized peel. According to^53^, the low lipid content of jaboticaba peel may bring benefits to human health since a large part of the population consumes hyperlipidemic diets, which contributes to the emergence of obesity to many chronic diseases.

The protein content found was 6.13 g 100 g^−1^ and is similar to the value found by^51^ of 5.23 g 100 g^−1^. In^53^ evaluated the influence of drying temperature and obtained a crude protein content of 6.58, 6.06, and 5.95 g 100 g^−1^ at temperatures of 30, 45, and 60 °C, respectively.

The crude fiber content of jaboticaba peel powder was 4.36 g 100 g^−1^. In^54^ found for the fresh peel the value of 1.84 g 100 g^−1^ and for the lyophilized peel, 3.89 g 100 g^−1^, which is lower than the value found in the present work. Carbohydrates were the macronutrient with the highest content fraction on JPP (51.77 g 100 g^−1^). In^50^ found a lower value of 58.70 g 100 g^−1^. Since fiber content is included in total carbohydrates, the method used for carbohydrates estimation may be overestimating its value.

The neutral detergent fiber content found was 12.11 g 100 g^−1^, higher than the 5 g 100 g^−1^ found by^49^. In^55^, while characterizing the jaboticaba fruit and its byproducts, found insoluble fiber content of 27.03 g 100 g^−1^ and 26.43 g 100 g^−1^, for the Paulista and Sabará variety, respectively. The acid detergent fiber content represents the soluble fraction of total fibers. The acid detergent fiber content found was 12.32 g 100 g^−1^, whereas^50^ obtained a value of 20 g 100 g^−1^ of soluble fibers.

Anthocyanins are extremely unstable to environmental conditions such as light, pH, oxygen, temperature, and the presence of metal ions that can affect their stability^56^. The jaboticaba peel powder produced in the present work presented relevant anthocyanin contents of 83.59 mg 100 g^−1^. In^57^ found average values of total anthocyanins ranging from 4.9 to 147 mg 100 g^−1^ at five maturation stages. The authors also found that anthocyanin concentration increased with the maturation stage. Jaboticaba peel powder presented a flavonoid content of 108.91 mg 100 g^−1^, a lower value than the one found by^34^, which was 147.0 mg 100 g^−1^ for jaboticaba (peel + pulp) in natura.

The total carotenoid content found in jaboticaba peel powder (JPP) was 18.67 μg g^−1^ lycopene or 1.867 mg 100 g^−1^. In^34^, in their study with 18 non-traditional Brazilian fruits, including jaboticaba. The authors obtained for the fresh fruit (peel and pulp) a carotenoid content of 0.32 mg 100 g^−1^. Thus, the present work's value was higher, which can be justified by a higher presence of carotenoids on the peel than the pulp. Alternatively, the dehydration concentrated the carotenoids, and thus the value is higher.

The value of total phenolic compounds found for (JPP) in the present study was in the range of 2549.65mgGAE 100 g^−1^ after extraction for aqueous extract. In^58^ found for lyophilized jaboticaba peel 3612.00mgGAE 100 g^−1^ and considered it a good source of bioactive compounds. In^59^ found for jaboticaba powder a value of 2149.58mgGAE 100 g^−1^. In^50^ characterizing both the fresh jaboticaba whole fruit and peel, presented a total phenolic content of 11.400 and 3.215mgGAE 100 g^−1^, respectively.

The value obtained for antioxidant activity by the ABTS^•+^ method in the present work was 403.51 µmol Trolox g^−1^ for jaboticaba peel powder (JPP). In^34^ presented an antioxidant activity of 37.5 and 317 µmol Trolox g^−1^, for fresh and dried jaboticaba, values lower than those found for (JPP). The value found for the antioxidant activity for jaboticaba peel powder (JPP) by the DPPH^•^ method expressed by the EC50, after aqueous extraction, was 180.58 g g^−1^. A similar was found by^34^ with 138 g g^−1^ DPPH^•^ in dry matter using three consecutive extractions with organic solvents (methanol, acetone, and water) to obtain a final aqueous extract. Climatic and soil conditions affect grapes' phenolic composition since these factors influence their biochemical synthesis and, consequently, their biological properties^60,61^.

### Pasting and rheological properties

Table 2 presents the data on the sizing properties of the modified native red rice (SN) starch paste (MS1 and MS2) and their mixtures prepared with the jaboticaba shell powder.

**Table 2:** Pasting properties of native red rice starch paste (SN), modified (MS1 and MS2) and their mixtures prepared with jaboticaba peel powder.

Pasting properties						
Parameters (mPa s^−1^)	SN	MS1	MS2	SN + JPP	MS1 + JPP	MS2 + JPP
PV	3688 ± 259.26^B^	5164 ± 38.81^A^	3091 ± 68.73^C^	3475 ± 28.58^B^	4010 ± 48.58^D^	2788 ± 43.51^E^
MV	2399 ± 96.14^B^	3269 ± 86.36^A^	2527 ± 44.63^B^	2148 ± 66.51^C^	3050 ± 78.13^D^	2198 ± 39.37^C^
BD	1289 ± 284.72^B^	1894 ± 124.62^A^	564 ± 32.05^C^	1327 ± 39.95^B^	960 ± 32.36^D^	589 ± 18.04^C^
SB	2837 ± 142.57^A^	2799 ± 405.29^AC^	1705 ± 78.25^B^	2378 ± 86.03^C^	1060 ± 108.13^D^	1682 ± 22.18^B^
FV	5236 ± 233.69^A^	6068 ± 723.42^A^	4232 ± 106.51^B^	4526 ± 43.66^C^	4732 ± 35.35^D^	3258 ± 49.05^E^
Pasting temperature (°C)	79.90 ± 0.13^C^	80.71 ± 0.06^B^	82 ± 1.26^AB^	78.83 ± 0.83^D^	80.90 ± 0.43^B^	83 ± 1.45^A^
Peak time (min)	6.15 ± 0.15^A^	5.16 ± 0.10^B^	6.13 ± 0.13^A^	6.11 ± 0.07^A^	5.91 ± 0.10^A^	6.11 ± 0.03^A^

*PV* Peak viscosity, *MV* Minimum viscosity, *BD* Breakdown, *SB* Setback, *FV* final viscosity.

Different capital letters indicate a significant difference (*p* < 0.05) between formulations for each parameter analyzed *SN* native starch, *MS1* Modified starch by α-amylase, *MS2* Modified starch by α-amylase and amyloglucosidase, *JPP* Jaboticaba peel powder.

According to^61^, the highest values of peak viscosity or maximum viscosity were associated with a high proportion of ungelatinized starch, while the lowest values of viscosity were related to more considerable degradation gelatinization of starch. This degradation is generally attributed to the depolymerization and molecular entanglement resulting from the starch modification. The highest peak viscosity value was found for enzymatically modified starch 1 (MS1) with 5164.61 mPa.s, while the formulations SN and SN + JPP presented no significant differences. When comparing the mixtures' other values with the samples without jaboticaba peel powder, it was observed that for MS1 + JPP and MS2 + JPP, the values were lower. In^62^ found an increase in peak viscosity due to pre-gelatinized starch to rice flour for dumpling production, ranging from 4716 to 5036 mPa.s.

The minimum viscosity value was determined by the final viscosity minus the tendency to retrograde. No significant difference between the formulations SN and MS2 was found for minimum viscosity, revealing that the two enzymatic hydrolyzes were not enough to change this parameter if compared to native starch. The same trend was found for their respective mixes SN + JPP and MS2 + JPP, which shows that jaboticaba peel powder also did not change this pasting property. However, results reveal that the minimum viscosity for (MS1) presented a higher value with or without the addition of jaboticaba peel powder (JPP). In^63^ observed no significant difference for minimum viscosity during the production of starchy rice balls. In^64^ found differences between wheat starch incorporated with different proportions of barley starch citrate, obtaining values ranging from 610 to 1075 mPa.s.

Breakdown viscosity is caused by the disruption of gelatinized starch granules when a high shear rate combined with temperature is applied, causing a sudden viscosity loss^65^. The addition of jaboticaba peel powder in the SN and MS2 tests did not influence this parameter but reduced the breakdown in the MS1 + JPP. The higher the breakdown viscosity, the stronger the starch gel. The viscosity drop value is the peak viscosity value minus the minimum viscosity value^66^. The highest values for the viscosity drop were obtained for the formulations MS1 and SN + JPP, followed by native starch (SN).

Table 2 shows that samples SN and MS1 have a greater tendency to retreat (setback), with values of 2837.72 and 2799.15 mPa.s, followed by the sample (SN + JPP). MS2 and MS2 + JPP samples presented similar results, with no significant difference, according to Tukey's test with (*p* > 0.05). The addition of jaboticaba peel powder (JPP) decreased the retrogradation tendency primarily for MS1.

SN and MS1 presented the highest values for the final viscosity with significant differences comparing to the other formulations. Final viscosity can be related to the textural firmness parameter. The firmness values for the enzymatically modified starch 1 (MS1) and native starch (SN) formulations are similar. Alternatively, a different characteristic is observed when the texture profile is correlated to the rheological profile of jaboticaba peel powder (JPP) mixes. In^62^ obtained 2960 to 2969 mPa.s for glutinous rice flour with pre-gelatinized starch.

Table 2 shows the main rheology parameters for native (SN), enzymatically modified (MS1 and MS2) starches and their respective mixtures (SN + JPP, MS1 + JPP, and MS2 + JPP). No significant difference between the samples MS1 and MS2 and their respective mixtures MS1 + JPP and MS2 + JPP was found for the pasting temperature. The highest temperatures of paste formation or gelatinization were found for the mix MS2 + JPP and MS2. The jaboticaba peel powder had no interference in the paste formation temperature. In^63^ also found no changes in the pasting temperature. In^67^ studying rice and tapioca starch obtained pasting temperatures of 91.43 °C and 71.67 °C, respectively. In^68^ found variations in pasting temperature from 72.5 to 79.3 °C for quinoa flour.

Furthermore, only MS1 presented a significant difference for peak viscosity time than the other formulations, with values ranging from 5.16 to 6.15 min. This time is shorter than that found by^67^ for rice flour, 6.78 min.

Observing the rheological behavior of red rice starch paste and jaboticaba peel powder through Table 3, it is found that the shear stress increases with the increase of the deformation rate. This result indicates non-Newtonian fluid behavior, specifically pseudoplastic or shear-thinning. The same profile was found by^68^ in their studies with quinoa.

**Table 3:** Regressed coefficients for Ostwald-de-Waele model (Power Law) and texture parameters at 25 °C.

Ostwald-de-Waele model (power law)					
Formulations		Consistency index (K)	Flow Index behavior (n)	R^2^ (%)	MSD
SN	70 °C	17.00	0.42	99.71	1.21
MS1		38.15	0.31	99.87	0.94
MS2		27.02	0.36	99.84	0.41
SN + JPP		30.49	0.31	99.96	0.33
MS1 + JPP		30.95	0.26	99.95	1.04
MS2 + JPP		30.09	0.26	99.74	0.44
SN	25 °C	54.90	0.25	99.61	1.50
MS1		52.67	0.33	99.85	1.58
MS2		26.93	0.38	99.82	0.71
SN + JPP		32.99	0.31	99.91	0.52
MS1 + JPP		37.10	0.25	99.90	1.26
MS2 + JPP		33.66	0.28	98.84	0.77

Texture parameters					
Formulations	Firmness (N)	Gumminess (N)	Cohesiveness	Adhesiveness (N.m)	
SN	0.34 ± 0.01^C^	0.28 ± 0.01^C^	0.85 ± 0.01^A^	0.58 ± 0.01^C^	
MS1	0.33 ± 0.01^C^	0.30 ± 0.01^C^	0.83 ± 0.01^B^	0.43 ± 0.01^E^	
MS2	0.29 ± 0.00^D^	0.23 ± 0.01^D^	0.81 ± 0.02^BC^	0.54 ± 0.00^B^	
SN + JPP	0.47 ± 0.02^A^	0.39 ± 0.02^A^	0.84 ± 0.01^A^	0.73 ± 0.02^A^	
MS1 + JPP	0.37 ± 0.01^B^	0.35 ± 0.01^B^	0.85 ± 0.01^A^	0.33 ± 0.01^F^	
MS2 + JPP	0.33 ± 0.01^C^	0.27 ± 0.01^C^	0.80 ± 0.01^C^	0.48 ± 0.01^D^	

Equal overlapping capital letters in the same column did not differ significantly between the formulations studied (*p* > 0.05).

*SN* native starch, *MS1* Modified starch by α-amylase, *MS2* Modified starch by α-amylase and amyloglucosidase, *JPP* jaboticaba peel powder, *MSD* mean square deviation.

The pseudoplastic behavior can be explained by the progressive orientation of soluble starch molecules in the flow direction and the rupture of the intra-and intermolecular bonding system in the starch network that increases shear force sensitivity^69,70^. Dynamic viscoelastic properties can be used to describe three-dimensional starch and hydrocolloid network structures^71^. Generally, the contribution to intermolecular interaction in the final gel formation of the mixture depends on two main factors: (1) the appropriate concentration and (2) the intermolecular synergistic effect, which is affected by molecular conformation, molecular weight, and starch structure^72,73^.

Table 3 presents the parameters of the Ostwald-de-Waele rheological model, fitted to the experimental data for the different formulations and temperatures studied, and the coefficients of determination (R^2^) and the mean square deviation (MSD).

The fluid behavior index in the Ostwald-de-Waele model was found to be less than 1 for all samples. The same behavior was found by^62^ for the mixture between wheat starch with barley starch citrate and^69^ for cornstarch. The consistency index (K) presented lower values at 70 °C, except for (MS2), indicating temperature dependence. The native starch formulations presented the most considerable difference between the temperatures of 25 °C and 70 °C.

### Texture analysis

Table 3 shows the texture measurements for the native starch (SN) and the modified starch (MS1 and MS2) formulations, as well as their respective mixtures with 10% jaboticaba peel powder (SN + JPP, MS1 + JPP, and MS2 + JPP). The firmness of the enzymatically modified starch paste with α-amylase and amyloglucosidase (MS2) was lower compared to the starch paste modified by the α-amylase enzyme (MS1) and native starch (SN). In^73^ obtained firmness results similar to (MS2) since the starches modified by acid hydrolysis presented lower firmness when compared to the native starch. In the present work, only after the addition of jaboticaba peel powder (SN + JPP, MS1 + JPP, and MS2 + JPP), this desired characteristic was obtained.

In^74^ obtained cake with softer texture when native cornstarch was replaced by hydrolyzed and acetylated starch. This finding shows that modification of starch forms a smoother paste, and smoothness increases as the number of modifications applied to a native starch increases. However, this did not happen in the formulation (MS1) because the enzymatic hydrolysis process using α-amylase selectively attacks the amylose present in the starch. Subsequently, the amylopectin, mainly responsible for the texture characteristics, was not affected during the first hydrolysis. Moreover, in the hydrolysis second step, now with amyloglucosidase, the hydrolysis percentage was higher, and thus differences in firmness were found.

According to^76^ reported that the addition of mango peel powder on bread production prevents the formation of a three-dimensional network structure, increasing the product firmness^77,78^. Firmness increase is generally a phenomenon observed when the fiber is added to products^79^.

In^80^ define gumminess as the energy required to disintegrate a semi-solid food. The native red rice starch presented a gumminess value of 0.28 N, which is higher than formulation MS2 (0.23 N) and similar to MS1 (0.30 N). All starch + jaboticaba mixtures presented higher firmness and gumminess than the formulations (SN, MS1, and MS2). This result confirms that the jaboticaba peel is rich in fibers, which gives the paste more support when gelatinized.

The cohesiveness of the native starch paste was higher than the modified starch paste (MS1 and MS2). This result suggests that the higher the number of hydrolysis steps, the lower the starch paste value's cohesiveness. However, the only significant difference noted for this parameter was between native starch (SN) and its respective mixture (SN + JPP), in which an increase in cohesiveness was noticeable. In^81^ obtained corn starch cohesion values ​​of up to 0.8, close to that obtained in the present study. In^67^ obtained values of 0.23 for rice flour and 0.90 for tapioca starch. In^76^ observed that the bread cohesiveness significantly decreased with increasing mango peel powder. Cohesiveness is a parameter that reflects the damage tolerance and sample integrity and is defined as the sample internal contraction force^23^.

All formulations presented a significant difference between them for the adhesiveness parameter. The sample SN presented higher adhesiveness than all the other starches without JPP but lower adhesiveness than the mixture with JPP. Inversely, the enzymatic modified starch 1 (MS1) presented lower adhesiveness than its respective mix (MS1 + JPP), which obtained the lowest adhesiveness of the study.

This result suggests that after starch pastes were heated, the intermolecular bonds were broken, and new bonds with the water molecules were formed, thereby increasing its adhesiveness. In^75^ observed that mango peel powder's addition did not influence adhesiveness, unlike what was found for jaboticaba peel powder in the present study.

### Water, oil, and milk absorption

Water, oil, and milk absorption profiles for all formulations are found in Table 4.

**Table 4:** Water, oil and, milk absorption capacity of all formulations.

Absorption Capacity (g 100 g^−1^)			
Formulations	Water	Oil	Milk
SN	50 ± 2.71^E^	47 ± 3.13^D^	50 ± 2.46^C^
MS1	58 ± 2.68^D^	56 ± 2.35^C^	65 ± 3.67^AB^
MS2	79 ± 3.34^B^	57 ± 2.67^C^	60 ± 1.89^B^
SN + JPP	66 ± 2.14^C^	57 ± 2.14^C^	63 ± 1.78^B^
MS1 + JPP	82 ± 1.12^B^	65.9 ± 0.98^B^	72 ± 1.45^A^
MS2 + JPP	90 ± 1.05^A^	71 ± 1.85^A^	70 ± 2.15^A^

Different capital letters indicate a significant difference (*p* < 0.05) between formulations *SN* native starch, *MS1* Modified starch by α-amylase, *MS2* Modified starch by α-amylase and amyloglucosidase, *JPP* jaboticaba peel powder.

The ability of modified starches to absorb water, oil, and milk is higher when compared to native starch. This result suggests that water absorption is related to the paste's cold viscosity since only damaged starch granules absorb water at room temperature. The absorption mechanism is explained by binding water molecules to hydrophilic groups (–OH), resulting in gel formation with a higher viscosity^82^. On average undamaged starch absorbs 33% of its weight in water^82^. The difference between the absorption of different components was more significant for the formulation (MS2) in water absorption than the other formulations, while for the formulation (MS1), no significant differences were observed between oil and milk absorption.

In^83^ obtained as a result (88.17; 97.12; 82.04%) for water absorption and (80; 73; 74%) for oil absorption before a freeze–thaw cycle, respectively for corn, wheat, and tapioca starch. In^64^ found water absorption values of 98% for wheat starch and values ranging from 73 to 89% for wheat starch mixed with different proportions of barley starch citrate. In^84^ found that oil absorption was higher than water absorption for native and gamma-irradiated wheat starch. In^83^ found that the addition of pre-gelatinized starch on rice balls decreased, from 98.34 to 93.73%, the percentage of immobilized water, indicating that pre-gelatinized starch has a stronger water absorption and retention capacity.

The addition of jaboticaba peel powder to the starches contributed to increased water, oil, and milk absorption capacity. The highest absorption for water and oil was found for the MS2 + JPP formulation. However, no significant differences were found between MS1 + JPP and MS2 + JPP for milk absorption. In^85^ found a range of 70.09 to 85.66% in oil absorption capacity for jackfruit seed starches. In^86^ obtained values for water-binding capacity ranging from 0.93 to 2.01 g water/g solid for the mix consisting of starchy rice flour and protein to produce gluten-free cookies, whereas, for oil absorption, the values ranged from 1.82 to 1.96 g oil/g solid.

The high water, oil, and milk absorption capacity of enzymatically modified red rice starch with the addition of jaboticaba peel powder demonstrates a desirable property for employing as an ingredient in food products requiring high water, fat, and milk retention. The obtained results suggest its best use as a thickener and stabilizer in fluids and emulsions.

### Syneresis index

Table 5 shows the syneresis index values after the 28-day storage.

**Table 5:** Syneresis index throughout the 28-day storage period.

Syneresis index (g 100 g^-1^)					
Formulations (g 100 g^−1^)	Days of storage				
	D0	D7	D14	D21	D28
SN	31 ± 2.41^Aa^	33 ± 1.41^Aa^	37.96 ± 0.89^Ab^	43 ± 1.41^Ac^	44.15 ± 0.51^Ac^
MS1	20 ± 2.29^Ba^	22 ± 1.29^Ba^	26 ± 2.29^Bb^	30.98 ± 0.59^Bc^	32 ± 1.09^Bc^
MS2	16 ± 1.12^Ca^	19 ± 1.03^Cb^	21 ± 1.12^Cb^	27 ± 1.14^Cc^	29 ± 1.10^Cc^
SN + JPP	25 ± 3.12^Ba^	27 ± 1.12^ Da^	29 ± 2.15^Ba^	35 ± 1.45^Db^	36 ± 0.78^Db^
MS1 + JPP	15 ± 1.04^CDa^	17 ± 1.04^CEa^	19 ± 1.02^CDa^	25.28 ± 0.44^Eb^	27 ± 1.01^Eb^
MS2 + JPP	13.01 ± 0.68^ Da^	15.22 ± 0.59^Eb^	18.08 ± 0.51^Dc^	**22.31 ± 0.61**^**Fd**^	23.11 ± 0.57^Fd^

Different superscript uppercase letters denote significant differences between formulations for the same storage day (p < 0.05). Different superscript lowercase letters denote significant differences between the storage days for the same formulation (p < 0.05).

*SN* native starch, *MS1* Modified starch by α-amylase, *MS2* Modified starch by α-amylase and amyloglucosidase, *JPP* jaboticaba peel powder.

Analyzing the formulations on the same day of storage, a significant difference between SN and enzymatically modified starches (MS1 and MS2), this behavior is confirmed for all storage days.

The addition of jaboticaba peel powder in the mixture's preparation helps decrease the syneresis index of the formulations MS1 + JPP and MS2 + JPP, which showed lower retrogradation on days D0 and D7. However, in the following days, the mixture MS2 + JPP obtained a significant difference and became the formulation with a lower value for this parameter. For MS2 and its respective mixture, the differences started at D7. These two formulations presented the lowest value for all storage days. MS2 + JPP was able to trap the water molecules avoiding further material retrogradation, and consequently, a lower syneresis rate was found for this sample at D28.

Syneresis results for formulations SN and MS1 obtained a significant difference only after D14, whereas their respective mixtures with JPP, only after D21 such significant difference was found. The amount of syneresis reflects the stability of starch in the freeze–thaw cycle. Additionally, the freeze–thaw stability is a critical feature in determining starch resilience to undesirable physical changes during freeze–thaw processes, which is also an indicator of starch retrogradation^68,87^.

In^70^ obtained for corn starch with the addition of different proportions of konjac-glucomannan, syneresis values ranging from 24.78 to 60.45%. In^84^, his study with native and irradiated wheat starch, the authors obtained syneresis values ranging from 0.77 to 46.66%, being the highest value found for native starch after 120 h of analysis, a value considered intermediate to that found in the present study. The increased percentage of syneresis may be attributed to macromolecules' association, particularly amylose, due to their linear structure and reinforced by amylopectin chains. During the freezing of a starch gel, the transformation of liquids into ice crystals increases the local concentration of starch molecules. This phenomenon accelerates intermolecular associations classified mainly as hydrogen bonding. When thawed, the ice turns to water and is likely to be released from the gel, producing a spongy texture. This phenomenon is known as syneresis^88^.

## Conclusions

Enzymatic hydrolysis of red rice starch using a two-step treatment with α-amylase and then amyloglucosidase caused rheological and textural alterations in paste formation. Fiber-rich jaboticaba peel powder inclusion on starch revealed to be a texture modifier. The firmness, gumminess, and final viscosity of starches increased with the inclusion of JPP, while the tendency to setback was reduced. Besides texture, JPP proved to be a source of anthocyanins and phenolic compounds with antioxidant power. A shear-thinning behavior was found for all samples, with JPP causing a further deviation from the Newtonian behavior. The addition of jaboticaba peel powder to starches increased water, oil, and milk absorption capacity, while syneresis remained stable over a 28-day storage period. According to the rheological and technological properties presented in each formulation, some applications can be suggested. For example, the modified starch can be used as a thickener or as an emulsifier or can even be applied as a humectant to increase water retention while lowering water activity. Simultaneously, the produced mixtures are appropriate in the food industry to be formulated in sponge cakes, bread, cookies, yogurts, and dairy beverages. Finally, considering all results, it is possible to conclude that the addition of jaboticaba peel powder had a positive effect on native and modified starches' technological properties and that its inclusion is a positive alternative for the development of new functional food products.
